# Detection and mapping of illicit drugs and their metabolites in fingermarks by MALDI MS and compatibility with forensic techniques

**DOI:** 10.1038/srep11716

**Published:** 2015-06-29

**Authors:** G. Groeneveld, M. de Puit, S. Bleay, R. Bradshaw, S. Francese

**Affiliations:** 1 Department of Fingerprint Research, Netherlands Forensic Institute, The Hague, Netherlands; 2 Biomedical Research Centre, Sheffield Hallam University, Sheffield, UK; 3 Technische Natuur Wetenschappen, Delft University of Technology, Delft, The Netherlands; 4 Centre for Applied Science and Technology, Home Office, Sandridge, UK

## Abstract

Despite the proven capabilities of Matrix Assisted Laser Desorption Ionisation Mass Spectrometry (MALDI MS) in laboratory settings, research is still needed to integrate this technique into current forensic fingerprinting practice. Optimised protocols enabling the compatible application of MALDI to developed fingermarks will allow additional intelligence to be gathered around a suspect’s lifestyle and activities prior to the deposition of their fingermarks while committing a crime. The detection and mapping of illicit drugs and metabolites in latent fingermarks would provide intelligence that is beneficial for both police investigations and court cases. This study investigated MALDI MS detection and mapping capabilities for a large range of drugs of abuse and their metabolites in fingermarks; the detection and mapping of a mixture of these drugs in marks, with and without prior development with cyanoacrylate fuming or Vacuum Metal Deposition, was also examined. Our findings indicate the versatility of MALDI technology and its ability to retrieve chemical intelligence either by detecting the compounds investigated or by using their ion signals to reconstruct 2D maps of fingermark ridge details.

Over the past seven years, Matrix Assisted Laser Desorption Ionisation Mass Spectrometry (MALDI MS) has proven to be a versatile analytical technology for gathering information (or intelligence) from latent fingermarks by detecting and mapping their chemistry^1^,^2^,^3^,^4^. However, despite a number of promising advances and its current evaluation in pseudo-operational trials in collaboration with the West Yorkshire Police (UK), much research is still needed to assess its full potential and the forensic scenarios in which this technology could be feasibly applied. Recent work undertaken on fingermarks deposited on a range of surfaces (porous and non-porous) has indicated varying degrees of MALDI MS compatibility with a number of forensic enhancement techniques (FET) conventionally used by Crime Scene Investigators (CSI) to visualise fingermarks, including aluminium and TiO_2_ powders, ninhydrin, 1,8-diazafluoren-9-one, cyanoacrylate Fuming (CAF) and Vacuum Metal Deposition (VMD)^5^. Compatibility ranged from the ability to detect molecular species after development (including endogenous species and some persistent chemicals present in toiletry products) to the opportunity to additionally map their distribution, thus providing multiple molecular images of the fingermark ridge pattern. Both levels of compatibility are of interest herein. The ability to image molecules in fingermarks using MALDI MS Imaging (MALDI MSI) may provide additional physical information (ridge detail) to support suspect identification^6^,^7^. Alternatively, following successful enhancement by a conventionally employed FET, the much faster MALDI MS Profiling (MALDI MSP) could be used to detect the chemistry of the marks to provide additional intelligence on the suspect’s lifestyle or actions prior to depositing the mark. In the aforementioned study^5^, VMD was the most compatible technique with MALDI MSI, most likely due to the reported ability of gold to act as a strong signal enhancer in mass spectrometry^8^. However, in these initial studies, attention was mainly focused on the detection and mapping of endogenous compounds. CAF on the other hand, although not the FET least compatible with MALDI, did not allow the retrieval of the same molecular species that were found following the application of VMD prior to MALDI MSI. Therefore, both the CAF-MALDI and VMD-MALDI workflows need further investigation to make the technology operational. These studies are particularly appropriate now, owing to the routine operational use of CAF and VMD and the recent interest from the scientific community in their compatibility with other analytical spectroscopic and spectrometric techniques. In addition to the work done by Francese’s group, VMD has been examined in conjunction with another analytical technique to provide intelligence at a molecular level by Bailey’s group^9^. In particular, they recently reported the application of a number of FET, including VMD, followed by TOF SIMS. The focus of this paper was mainly on demonstrating the ability to obtain TOF SIMS (molecular) images of developed marks where conventional FET had failed. Though this was demonstrated for a number of FET, no data/images were shown for a VMD-TOF SIMS treated fingermark. Compatibility with CAF has received more attention^9^,^10^,^11^. In particular, Day *et al.*^10^ considered a “handling” scenario and demonstrated the ability to detect a number of drugs in spiked ungroomed and CAF-developed fingermarks using Raman spectroscopy; the drugs investigated included codeine, cocaine, amphetamine, barbital and nitrazepam (as well as caffeine, aspirin, paracetamol, starch and talc, which can be used to adulterate drugs). Sundar and Rowell^11^ also considered a handling scenario for two drugs of abuse, specifically cocaine and methadone, in ungroomed fingermarks (in addition to caffeine, aspirin, and paracetamol). In this study, both SALDI MS and MALDI MS were investigated separately, in combination with CAF. Though the MS experiments performed are not very informative, especially because of the lack of confirmatory MS/MS analysis, there were indications that cocaine and methadone could be detected in un-lifted marks by both techniques. Moreover, the data suggested that only SALDI MS was successful in detecting these species in lifted marks following the exposure of CAF marks to acetone vapours prior to mass spectrometric analysis. It is, however, difficult to establish the actual significance of the reported SALDI images for cocaine and methadone in the CAF-developed marks as the images were highly pixelated (no ridge detail), not normalised and had no colour scale.

As there is only one reported study using MALDI MS for the detection of (only) two illicit drugs in fingermarks^11^, the argument for a more comprehensive study that investigates a larger range of drugs, as well as their metabolites, in developed marks is compelling. Additionally, as this previous study^11^ investigated these drugs only and in large amounts (1 mg), we wished to probe the sensitivity of MALDI technology by significantly lowering the amount of drug material. With respect to our previously published research, this is the first time that drugs and metabolites have been investigated by MALDI MSP and MSI as exogenous substances in undeveloped and pre-developed marks. This current research also provides insight into which drugs/metabolites are more amenable to MALDI MSI, particularly post-CAF and post-VMD development, as well as insight into the detection constraints and sensitivity limitations of the technology. Furthermore, this work has shed light on the most efficient FET-MALDI workflows (here FET refer to CAF and VMD). The latter point is very important and is the essence of all developmental work being undertaken in our laboratory to ensure that MALDI is used operationally and does not remain an academic exercise.

For this study, 17 compounds (parent drugs and metabolites, including the methamphetamine and 4-methylamphetamine isomers) were selected from 5 different classes of drug: amphetamines, alkaloids, opioids, cannabinoids and designer drugs. The inclusion of this number of species in these MALDI MS profiling and imaging analyses reflects the importance of detecting forensically relevant substances such as drugs of abuse and their metabolites in pre-developed marks. Mapping such “story telling” substances directly onto the identifying fingerprint ridges will potentially generate circumstantial, associative or even corroborative evidence on the suspect’s lifestyle and activities, allowing more informed criminal investigations and judicial debates. Importantly, detecting drug metabolites would indicate drug “abuse” rather than “handling”, which pertain to two very different forensic scenarios.

This study initially established the “basics” and the potential of MALDI for the detection of drugs and their metabolites in fingermarks. For this purpose, the ionisation efficiency was initially optimised in both MS and MS/MS modes (the latter for a confirmatory test on the presence of the substance). The limits of detection (LOD) were subsequently investigated, which informed subsequent fingertip spiking experiments. Two scenarios were tested to mimic “handling” (drug concentrations of the order of 0.5 μg of the pure compound contaminating the tip of a finger) and “abuse” (drug concentrations depended on the LOD obtained during preliminary studies and ranged from 0.05 to 0.0005 μg of pure compound). However, in the latter case, the literature offers no information on the possible amounts eventually retrievable in fingermarks. The only available studies refer to the quantitation of some of these species in sweat patches. For example, in a study by Barnes *et al.*^12^, the excretion of methamphetamine in sweat following consumption was demonstrated by GC-MS, with values ranging between 307 and 53.8 ng/patch, although no metabolites were identified. In another study by Barnes *et al.*^13^, the excretion of 3,4-methylenedioxy-methamphetamine (MDMA) was investigated using GC-MS. These analyses showed the presence of MDMA as the primary analyte, in concentrations up to 3007 ng/patch, and the formation of the methylenedioxyamphetamine (MDA) metabolite was detected in only 29.4% of the patches at a concentration lower than 172 ng/patch. A study by Kacinko *et al.*^14^ showed that following cocaine administration, cocaine was the primary analyte detected within sweat patches using GC-MS, with up to 375.4 ng/patch. Ecgonine methyl ester (EME) was detected more often, with up to 35.9 ng/patch, whereas benzoylecgonine (BZE) was recovered at levels up to 47 ng/patch. Kintz *et al.*^15^ showed that following heroin use, the intact drug is the primary analyte, with up to 96.3 ng/patch detected by GC-MS, though the primary metabolites of heroin, 6-monoacetylmorphine (6-MAM) and morphine, were also detected, with up to 24.6 and 11.2 ng/patch, respectively. Finally, as studied by Huestis *et al.*^16^, Δ9-tetrahydrocannabinol (THC, cannabis) is the primary compound observed in sweat patches, retrieved at a concentration of up to 3.85 ng/patch using GC-MS. Though the primary metabolite, THCA, was not observed, its detection was investigated here.

In the present study, to verify the possibility of retrieving and mapping drugs and their metabolites following the pre-development of drug-containing ungroomed fingermarks, marks were subjected to different types of CAF-MALDI, VMD-MALDI and CAF-VMD-MALDI workflows, with MALDI being employed in both profiling (MS and MS/MS) and imaging (MSI) modes. Findings from this study confirm previous observations indicating that VMD works much more compatibly with MALDI than CAF, with the ion signals appearing as strong or even stronger after VMD development than after no prior development. Within the CAF-MALDI workflow, the overall results indicate that although the drug signal intensity is much lower in many cases, as an effect of pre-development, intelligence can still be provided on the presence of these species. All drug and metabolite ion signals were confirmed through post-imaging MS/MS experiments, and on many occasions, they were also still intense enough to provide an image of the ridge detail. These results represent a further step towards the implementation of MALDI technology in forensic casework.

## Materials and Methods

All methods were carried out in accordance with the approved guidelines; specifically, methodologies and chemicals were risk assessed and approved by the Bioscience Technical Team (HWB Professional and Technical Services). The researcher performed the experiments using his own fingermarks, after washing fingertips as previously reported^1^. No ethical approval was required for this work, as the procedure for fingermark use in these studies is considered minor by Sheffield Hallam University Ethics Research Committee.

## Materials

Acetone, acetonitrile (ACN), ethanol (EtOH) and methanol (MeOH) were purchased from Fisher Scientific (Loughborough, UK). α-Cyano-4-hydroxycinnamic acid (α-CHCA) and trifluoroacetic acid (TFA) were purchased from Sigma-Aldrich (Poole, UK). Amphetamine (speed, AMP), methylbenzoylecgonine (cocaine, COC), diacetylmorphine (heroin, HER), Δ9-tetrahydrocannabinol (cannabis, Δ9-THC), 3,4-methylenedioxy-methamphetamine (ecstasy, MDMA), methylenedioxyamphetamine (MDA), ecgonine methyl ester (EME), benzoylecgonine (BZE), 6-acetylmorphine (6-MAM), morphine (MOR) and 11-nor-9-carboxy-THC (THCA) were purchased as analytical references from Cerilliant (Sigma-Aldrich, Zwijndrecht, The Netherlands). An illicit drug calibration mixture consisting of 2C-B, amphetamine, 4-methylamphetamine, methamphetamine, MDA, MDMA, MDEA, mCPP, cocaine, heroin and methadone was supplied by the Netherlands Forensic Institute, Department of Illicit Substances (The Hague, The Netherlands). MALDI target OPTI TOF spotless inserts were purchased from Applied Biosystems (Foster City, CA, USA). Double-sided conductive carbon tape was purchased from TAAB (Aldermaston, UK). ALUGRAM SIL G/UV254 pre-coated aluminium sheets were purchased from Boomlab (Meppel, Netherlands). Ethyl-2-cyanoacrylate for cyanoacrylate fuming was purchased from BVDA (Haarlem, The Netherlands).

### Instrumentation and instrumental settings

All mass spectrometric analyses were conducted using a modified Applied Biosystems “Q-Star” Pulsar *i* hybrid quadruple time-of-flight (QqTOF) instrument (Concord, Ontario, Canada). The “Q-Star” Pulsar *i* system consists of an orthogonal MALDI source that has been modified to incorporate a SPOT 10 kHz Nd:YVO_4_ solid-state laser (Elforlight Ltd., Daventry, UK)^17^ with a wavelength of 355 nm and a pulse duration of 1.5 ns that produces an elliptical spot size of 100 × 150 μm. MALDI MS spectra were obtained in positive ion mode in the mass range between m/z 50 and 1000. The declustering potential 2 was set at 15 arbitrary units (a.u.) and the focus potential at 10 a.u. with an accumulation time of 0.117 sec. MALDI MS/MS spectra were acquired after setting the declustering potential 2 at 15 a.u., the focusing potential at 20 a.u., the collision gas (argon) at 2 a.u. with the collision energy ranging between 15 and 35 a.u., according to the drug being analysed. The collision gas pressure was set at 12 a.u. Mass spectra were processed either in Analyst MDS Sciex V2 (Concord, Ontario, Canada) or converted to text files and processed using the open source multifunctional mass spectrometry software mMass^18^,^19^. Molecular images were acquired at a spatial resolution of 150 × 150 μm in raster mode using the oMALDI server 5.1 software supplied by MDS Sciex (Concord, Ontario, Canada) and processed using Biomap (Novartis, Basel, Switzerland). Cyanoacrylate fuming (CAF) was performed using a modified glass fish-tank equipped with an IKA Ret heater/stirrer set at 120 °C as the heating source, an aluminium fuming cupboard and a mounting system to enclose the aluminium slides on which marks were deposited. Suitable humidity was achieved by heating an aluminium bowl containing dH_2_O prior to the fuming of ethylcyanoacrylate. A humidity sensor was employed to ensure that a relative humidity of ~80% was achieved. Vacuum metal deposition (VMD) was performed at the Home Office Centre of Applied Science and Technology (CAST, UK) according to the procedure recommended by the Home Office edited Fingermark Visualisation Manual^20^.

### Drug/metabolite limit of detection (LOD), selectivity and confirmation of identity

The drug/metabolite standard solutions were diluted to a concentration of 10 μg/mL using MeOH and subsequently mixed thoroughly with 10 mg/mL α-CHCA in 70:30 v/v ACN:0.5% TFAaq at a ratio of 1:1. Aliquots of 0.5 μL were spotted separately onto a previously cleaned aluminium plate and subsequently subjected to MALDI MS and MALDI MS/MS experiments.

For the determination of LOD, ungroomed fingermarks were prepared as reported previously^1^. On top of each mark, a serial dilution containing a mixture of compounds at five pre-defined dilution levels was spotted. The standard mixtures studied were a) AMP, MDMA and MDA; b) COC, BZE and EME; c) HER, 6-MAM and MOR; and d) Δ9-THC and THCA. Every mixture was prepared in a range of compound concentrations: 10, 1, 0.1, 0.01 and 0.001 μg/mL, thus preparing 5 concentration points for each of the mixtures (a–d). For every standard mixture, 0.5 μL were spotted at five separate locations (at different compound concentrations) across the length of the mark. Upon drying under ambient conditions, the marks containing each of the 5 spots were spray-coated with 5 mg/mL α-CHCA in the 70:30 v/v ACN:0.5% TFAaq matrix solution. Four layers of matrix were applied using the SunCollect autospraying system (SunChrom GmbH, Friedrichsdorf, Germany) at a rate of 2 μL/min employing “slow raster” settings. MALDI selectivity was assessed by analysing the complex mixtures containing 10 of the 17 studied compounds. Spiked ungroomed fingermarks were prepared by mixing the parent drug and the metabolite either at “physiological ratios” (as indicated by the available literature) or in a 1:1 ratio. Specifically, cocaine, BZE and EME were used to spike a fingermark at a ratio of 5:1:1; the same ratio was used for heroin, 6-MAM and morphine. For this ratio, the amounts of each pure compound were 2.5, 0.5 and 0.5 μg, respectively; for amphetamine/MDA and THC/THCA, a ratio of 1:1 was employed with 2.5 μg-amounts of the pure compounds.

MS/MS analyses were employed (with no further treatment of the mark) in addition to MS analyses, and the acquisition parameters were optimised to confirm the presence and identity of the species through the recovery of molecular weight and structural information. These analyses were applied to the pure standards and to the spiked fingermarks in post-imaging acquisition.

### Preparation of spiked latent fingermarks with exogenous compounds: “handling” *versus* “abuse” scenarios

Ungroomed fingertips were prepared as previously described^1^. Aliquots (50 μL) of a 10 μg/mL stock solution of the illicit drug/metabolite of interest (equivalent to 0.5 μg of pure compound for each standard) were applied to a pre-cleaned glass microscope slide (high concentration = “handling scenario”). The solvent was allowed to evaporate under ambient conditions prior to extensively dragging the fingertip side to side onto the glass slide to transfer as much analyte as possible. The fingertip was subsequently contacted with a pre-cleaned aluminium sheet, producing a drug-spiked, ungroomed fingermark. In addition, a further set of ungroomed marks was generated by spiking the fingertip in the same manner with a 50-μL aliquot of the compound of interest at a concentration equivalent to the previously determined limit of detection (low concentration = “abuse scenario”).

### Compatibility studies: FET-MALDI MSI workflow

Six replicates of ungroomed fingermarks enriched with drugs of abuse were prepared. Each fingermark was then spiked with a mixture of illicit drugs containing (i) AMP, (ii) COC, (iii) HER and (iv) Δ9-THC. Fingermarks deposited on aluminium slides were produced after spiking fingertips using a 50-μL aliquot of a 25-μg/mL standard solution (equivalent to 1.25 μg of pure compound) of this mixture using the previously described method. The marks were subsequently cut in half (to produce ‘split marks’). One half was left undeveloped, and the other was developed using (i) CAF, (ii) CAF followed by exposure of the mark to acetone vapours, (iii) CAF followed by Basic Yellow 40 dye-staining, (iv) VMD, (v) CAF followed by VMD or (vi) CAF followed by Basic Yellow 40 dye-staining followed by VMD. The undeveloped and developed split marks were reassembled together on a MALDI target plate to form a full fingermark sample before being sprayed with matrix solution (5 mg/mL α-CHCA 70:30 v/v ACN:0.5% TFAaq, 4 layers at 2 μL/min) and subjected to MALDI MSI analysis. For CAF (i), 0.25 g of ethyl-2-cyanoacrylate were heated at 120 °C in an aluminium cup at approximately 65–80% relative humidity using an in-house developed fuming cabinet containing a heating source and an aluminium bowl filled with water. The samples were suspended in the cabinet and developed within 5–10 minutes. In a separate experiment, CAF-developed marks were also exposed to acetone vapours (ii); specifically, acetone (0.5 mL) was placed into an aluminium fuming cupboard and heated at 50 °C (near the boiling point of acetone). Additional Basic Yellow 40 dye-staining of CAF-developed marks (iii) was performed as follows: the CAF-developed half mark was sprayed with a Basic Yellow 40 (BY40) working solution (2 g/L in EtOH). Excess Basic Yellow 40 staining solution was washed away using deionised H_2_O, and the mark was allowed to dry under ambient conditions. VMD (iv) was performed at CAST (Home Office, St Albans, UK). For VMD workflows, split marks were either subjected to VMD and then to MALDI (iv) or preliminarily developed with either CAF or CAF followed by Basic Yellow 40 dye-staining prior to VMD enhancement at CAST (samples (v) and (vi), respectively). Images of all developed half marks were captured using a VSC4CX videospectral comparator (Foster and Freeman, Evesham, UK) under visible light.

## Results and Discussion

**Figure 1:**
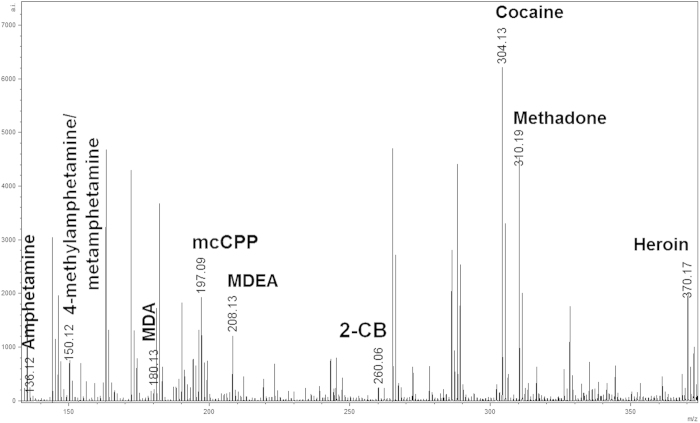
MALDI-QTOF-MS spectrum of a solution containing 10 illicit drugs at a concentration of 10 μg/mL mixed with an equal volume of matrix solution. Using the predominant [M+H]+ parent ions formed, the compounds can be identified based on MS and MS/MS experiments. Methamphetamine and 4-methylamphetamine were not differentiated in MS mode owing to mass-to-charge overlap (*m/z* 150.1).

As this is the first time that the detection and mapping of a vast range of illicit drugs and metabolites in fingermarks have been investigated by MALDI in a sequential workflow with forensic enhancement techniques (FET), the MS and MS/MS conditions were initially optimised. MALDI MS and MALDI MS/MS spectra were acquired in positive mode for 17 compounds, and optimised instrumental conditions were subsequently established. As shown in Table 1 and Fig. S1, all compounds were detectable as [M+H]^+^ ions in MS mode and generated characteristic ion fragment signatures in MS/MS mode. Furthermore, to provide insight into the specificity of MALDI detection, a mixture of ten compounds (5-dimethoxy-4-bromophenethylamine (2-CB), amphetamine, 4-methylamphetamine, methamphetamine, 3,4-methylenedioxyamphetamine (MDA), 3,4-methylenedioxy-N-ethyl-amphetamine (MDEA), meta-chlorophenylpiperazine (mCPP), cocaine, heroin and methadone) was analysed by MALDI MS. All ten species were detected, with no significant ion suppression impeding detection of any one species (Fig. 1). Although 4-methylamphetamine and methamphetamine, being isomers, have the same *m/z*, they were differentiated by MS/MS because of the difference in their fragmentation patterns, as Fig. S1 illustrates. In the MS analyses performed under optimised conditions, most compounds also exhibited the formation of in-source fragments (spontaneous fragmentation in the ion source of the mass spectrometer). In contrast for all other compound classes screened, in-source fragments of the amphetamine class showed a higher abundance than that of the corresponding molecular ion [M+H]^+^. In-source fragments can be a valuable signature for the rapid and more reliable identification of the drug/metabolite present in fingermarks, although MS/MS is always recommended; in fact, MS/MS data have been used throughout this study as a reference to definitively confirm the presence of the species of interest.

**Figure 2:**
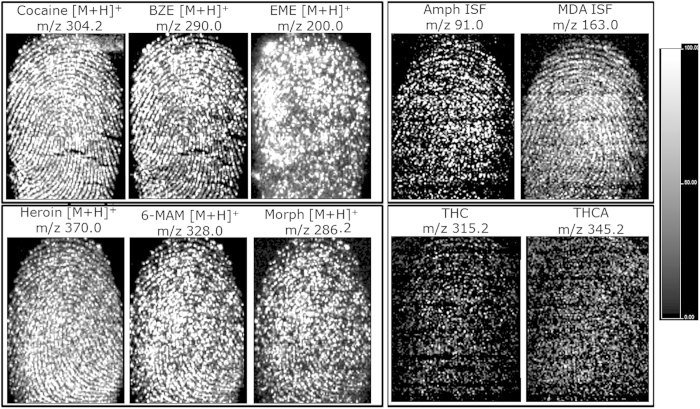
MALDI MS images of fingermarks simultaneously spiked with drugs and their metabolites at defined ratios to show the distribution of samples containing (**A**) cocaine, BZE and EME in a 5:1:1 ratio; (**B**) heroin, 6-MAM, morphine in a 5:1:1 ratio; (**C**) amphetamine and MDA in a 1:1 ratio; and (**D**) THC and THCA in a 1:1 ratio.

In subsequent experiments, fingertips were spiked with both the parent drug and its metabolites in either an approximate “physiological” ratio based on data from the available literature^14^,^15^, as for cocaine and its metabolites as well as heroin and its metabolites, or in an arbitrary 1:1 ratio, as for amphetamine/MDA and THC/THCA, to provide information on any potential ion suppression by the parent drug or the metabolite when using MALDI MSI. The molecular image of cocaine shows better ridge continuity than that obtained for BZE, though ridge detail can be fully obtained for the latter (Fig. 2). Conversely, EME did not show full ridge detail but rather a dot-like distribution. The molecular images of metabolites 6-MAM and morphine were slightly poorer than the one obtained for heroin, though they still provided ridge detail. Within these two classes of analytes, no evidence of ion suppression was found, but the higher concentration of the parent drug certainly improves the quality of the corresponding molecular images. MALDI MSI of 1:1 dual spiked marks of AMP/MDA and THC/THCA allowed for the detection and mapping of the species, but the quality of the images was very poor for THC and THCA. The poor THC/THCA image quality was observed previously in analyses in which these species were investigated separately; thus, it cannot be directly ascribed to the simultaneous presence of both species. Post-imaging MS/MS analyses were used to confirm the presence of all species investigated. All of these data show good drug screening capabilities in fingermarks containing multiple drugs (in a single analysis) as well as the capability to detect and map both the parent drug and its metabolites.

**Figure 3:**
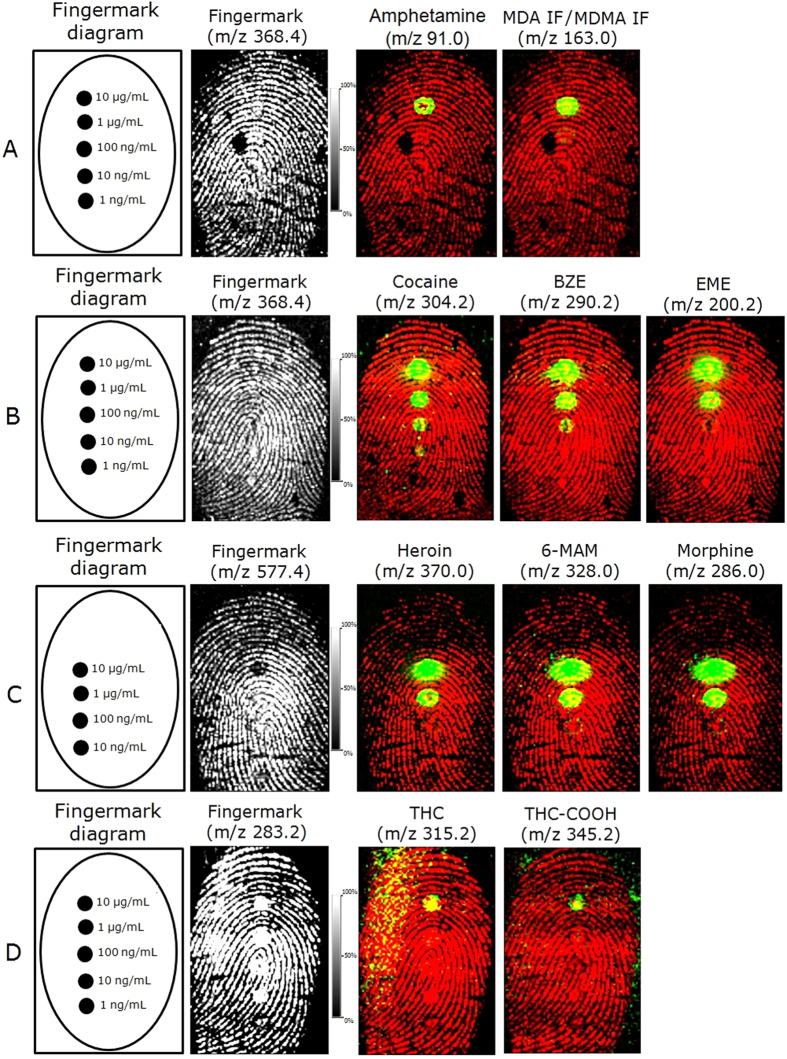
MALDI MSI of serial dilutions ranging from 10 μg/mL to 1 ng/mL of the different drug classes (drug/metabolites) spotted on top of a fingermark and subsequently spray-coated. LODs were determined from (**A**) the amphetamine mixture, (**B**) the cocaine mixture, (**C**) the heroin mixture and (**D**) the THC mixture. The ion signals of the compounds are shown as an overlay (green) on top of the ion signal originating from an endogenous species (red) depicting the ridge pattern.

Prior to the FET-MALDI workflow experiments, the limits of detection for MALDI had been established for a range of drugs/metabolites by investigating fingermarks spotted with serial dilutions of these compounds ranging from 10 to 0.001 μg/μL. Five spots of the drug/metabolite mixtures from each drug class were deposited across the length of the mark in a serial dilution. The mark was then spray coated with matrix and subjected to MALDI MSI analysis. The LOD recovered here refers to the concentration of drug/metabolite in the spot corresponding to the lowest dilution that could be observed in the processed molecular images of the compound of interest, and these values are reported in Table 1. As shown in Fig. 3 and Table 1, the LOD is strongly class and compound dependent, which is due to differences in their ionisation efficiencies. The amphetamine and cannabinoid classes show the lowest ionisation efficiencies with drugs and metabolites and could provide spot images at only the highest concentration tested (10 μg/mL). Alkaloid detection in spot images ranged from 10 ng/ml to 10 μg/mL, whereas the LOD for opioids was between 100 and 10 ng/mL. Further processing of the data by brightness-saturation of the images showed molecular images of some species in additional spots (Fig. S2). For example, MDA/MDMA were also visible at 1 μg/mL, and EME, heroin, 6-MAM and morphine were visible at 100 ng/mL. Notably, in all cases illustrated in Figs 3 and S2, it may be possible to obtain molecular maps of the drugs at concentrations between that corresponding to the last visible spot and that of the first spot not detected; however, this intermediate concentration will have to be determined. Additionally, in some cases, even if the spot was not visible as an image, the ion signal in the corresponding extracted spectrum was still detectable, though confirmatory MS/MS experiments were not performed. With these types of MALDI MSI experiments, the amount of each compound is spread within a defined circular area (the spot). Therefore, it is more appropriate to report LOD as a function of the amount of drug divided by its occupied area. By doing this, the observed LOD *for visible spots* was achieved as low as 0.009 ng/cm^2^.

**Figure 4:**
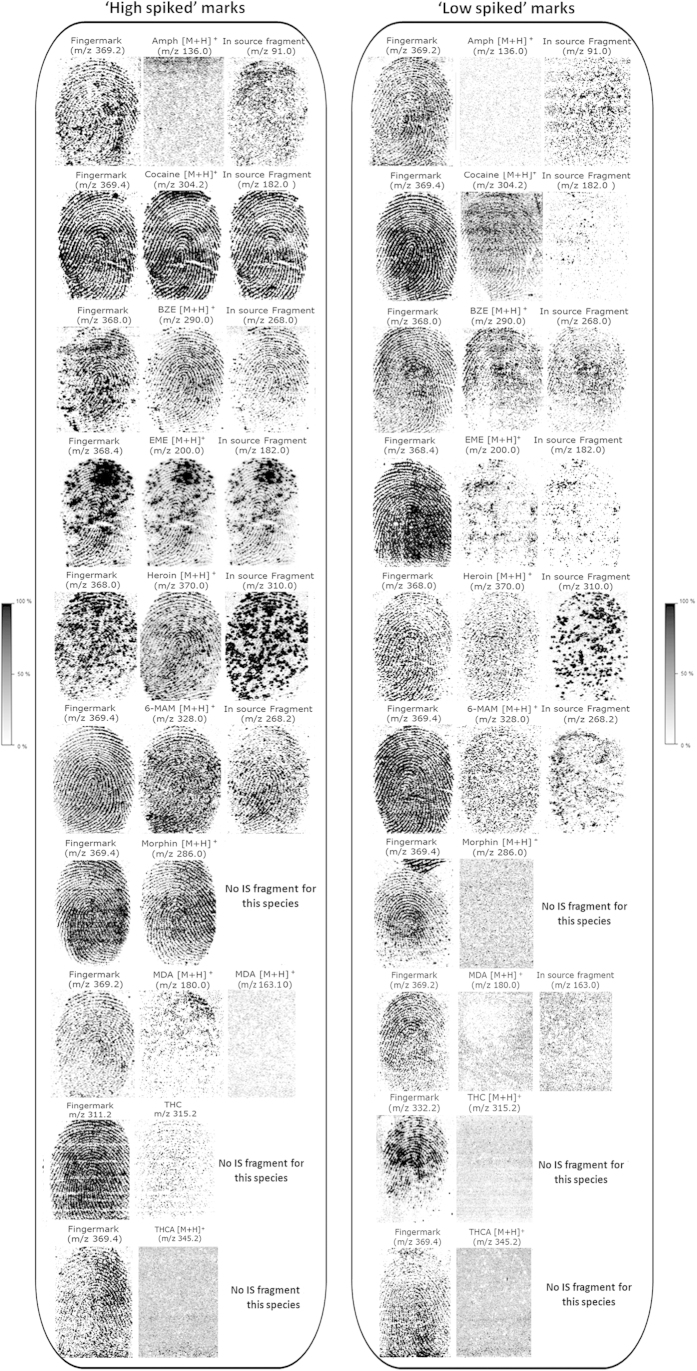
MALDI MS images of ‘high’ (“handling” scenario) and ‘low’ (“abuse” scenario) spiked fingermarks contaminated with amphetamine, cocaine, BZE, EME, heroin, 6-MAM, morphine, MDA, THC and THCA. All images were normalised against the matrix peak at *m/z* 190.

Further “sensitivity” experiments were conducted that took the data from previous LOD determinations into account. “Handling” and “abuse” scenarios were investigated by entirely spiking fingertips with either “high” (0.5 μg) or “low” drug/metabolite amounts (ranging from 0.0005 to 0.05 μg, respectively) prior to analysis by MALDI MSI. In similar studies investigating the detection of drugs in fingermarks, powder and/or tablets were used to transfer the drug onto the fingertip^10^,^21^. The amount transferred onto the fingertip or the amount of powder was not indicated in these studies; however, from the description, it can be speculated that milligram(s) amounts were transferred as a result of pure powder handling. Therefore, specifically transferring between 0.0005 and 0.5 μg of the drug/metabolite is a better approach for testing the sensitivity of the technology. Fingertips were spiked with ten selected drugs/metabolites individually (one compound per fingermark). Findings from this experiment are illustrated in Fig. 4. For the “handling” scenario in particular, the intensities of both parent ion and in-source fragments (when available) were high enough to generate molecular images of the compounds investigated, allowing reconstruction of the fingermark ridge pattern with second-level detail (*minutiae*) and grading from 1–4 according to the 0–4 scale^22^. For MDA and THC, partial fingermark images could be obtained, whereas no images were recovered for THCA; this reflects that the ionisation efficiency of this compound is lower than that of the other drugs investigated, as previously observed (Fig. 3). The ion signals from the drugs of interest show comparable (cocaine, EME, 6-MAM, morphine) or even better (BZE, heroin) images of the ridge pattern compared to the best image obtained from an endogenous compound, which is merely shown as a comparison (and therefore its identity is unimportant). In the “abuse” scenario, cocaine, BZE and heroin could be suitably mapped on the ridges, whereas partial and or discontinuous ridge detail was obtained for EME and 6-MAM. No images were recovered for morphine, MDA, THC and THCA, and little was visible for the in-source AMP fragments. It is possible to determine the amount of drug per area within the fingermark as both the concentration and the volume of the exogenous compound are known. Overall, within the “handling” scenario, MALDI MSI of a ~190 ng/cm^2^ fingermark results in chemical images depicting the pattern of the mark, including second level detail or showing the distribution of the drug/metabolite within the mark. Within the “abuse” scenario, the MALDI drug/metabolite molecular images generally depict a speckled ridge pattern, with the exception of cocaine, BZE, EME and heroin (albeit with different grades of ridge coverage). For these species, fingermark images could be retrieved at low concentrations of 0.19 (cocaine) and ~1.9 ng/cm^2^ (BZE, EME, heroin). For both the “handling” and the “abuse” scenarios, partial images could still be important. First, if a previously applied FET provides a good image for suspect ID and MALDI compatibility is proven, intelligence could still be provided by confirming the presence of these substances. Furthermore, partial images could still be superimposed onto the molecular images of other species present in the mark (such as endogenous ones) to demonstrate association of the drug/metabolite with the ridges of the suspect. Importantly, for full, partial or no fingermark molecular images, post-imaging MS and MS/MS profiling revealed and confirmed the presence of all investigated compounds (data not shown). Table 1 presents a summary of the MS and MS/MS detection of the drugs as well as the LODs calculated in ng/cm^2^ for the two types of imaging experiments performed (spotted marks and spiked marks within the “handling”/“abuse” scenarios). Notably, for the “abuse” scenario, the real excreted quantity that might be present in fingermarks is not known, but a study in which the authors have recently participated indicated the ability of MALDI MSP to detect BZE in fingermarks from patients in a drug rehabilitation clinic at levels corresponding to <16 ng/mL in oral fluids^23^.

**Figure 5:**
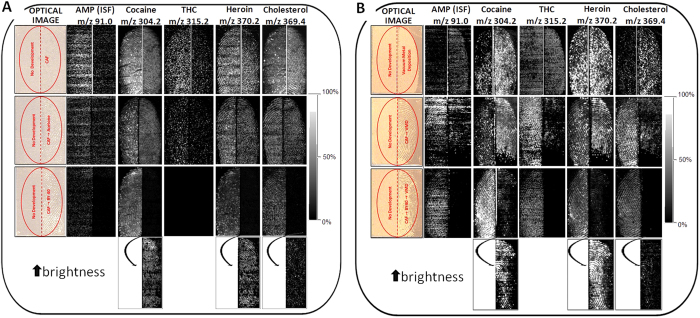
MALDI MS images of split fingermarks spiked with a mixture of 4 drugs and analysed with and without prior FET development. All images were normalised to total ion current, but each entire fingermark image was adjusted to optimal brightness and contrast. **A**: MALDI MS images acquired after CAF, CAF + acetone vapour exposure and CAF + BY40 staining. The last row of the panel shows the brightness increased to reveal enhanced 2D maps of cocaine, heroin and cholesterol. **B**: MALDI MS images acquired after VMD, VMD + CAF and CAF + BY40 staining + VMD. For both panels A and B, the last row of the panels shows brightness increased to reveal enhanced 2D maps of cocaine, heroin and cholesterol.

Information retrieved through confirmatory and LOD studies enabled the subsequent design of experiments to investigate the MALDI detection and mapping of a mixture of amphetamine, cocaine, heroin and THC in marks (a total of 1.25 μg spread across the mark area) that were developed with CAF and/or VMD prior to MALDI MSI using different workflows. Specifically, two sets of experiments were performed on split fingermarks, and in all experiment sets, one half of the mark remained undeveloped and was directly subjected to MALDI MSI as a reference sample. In experiment set A, spiked half marks were subjected to 3 types of CAF enhancement prior to MALDI MSI analysis: (i) CAF, (ii) CAF + acetone vapour exposure and (iii) CAF + BY40 staining. These types of enhancements followed by MALDI MSI were selected to determine which conditions allowed recovery of ridge detail and ion signals. Treatment with acetone following CAF was evaluated as it was recently reported to enhance the recovery of cocaine and methadone due to the partial dissolution of the CAF polymer^11^. Staining with BY40 following CAF was selected as this is the process often used in crime labs and is recommended to improve ridge enhancement in the CAST manual^20^. Molecular maps of the 4 drugs and cholesterol (the latter as an example endogenous compound recovery) are reported in Fig. 5A. Overall results corroborate previous observations regarding the ionisation efficiency of amphetamine and THC, which yielded the poorest molecular images. Amphetamine molecular maps were highly speckled both in the absence and presence of CAF development, although the ion signal appeared to be stronger in the undeveloped half of the mark. Treatment with acetone did not improve the quality of the image and BY40 staining did not yield any image. THC provided better ridge detail in one of the three undeveloped mark halves, but no image was obtained for the split mark analysed with condition (iii). Because the same image was theoretically expected for all of the undeveloped halves, the THC molecular maps lacked reproducibility, most likely due to the very low efficiency of ionisation of the compound and the strong dependence on the quality of the matrix co-crystallisation. Further investigations into different matrix systems and/or the employment of negative ionisation are needed, particularly for THC. Moreover, for THC, acetone treatment did not improve the quality of the image. Given that no THC image was obtained for the undeveloped half, it is not possible to comment on the effect of BY40, though image improvement is not expected. Cocaine and heroin behaved very similar to one another, as it was possible to obtain images for both undeveloped and developed marks under all conditions tested. In particular, though full ridge detail could be obtained following CAF and CAF-acetone development, sharper contours were observed for the corresponding undeveloped fingermark halves. BY40 had a marked effect on the intensity of the cocaine and heroin signals as faint ridge details were obtained for the CAF-BY40 treated fingermark halves compared to the untreated ones. However, it must be considered that brightness of the overall mark image was adjusted primarily to neatly observe the ridge detail in the undeveloped half; thus, a marked increase in the brightness shows that ridge detail was recoverable for cocaine, heroin and endogenous cholesterol also after BY40 staining (last row of Fig. 5A). The endogenous cholesterol behaved in the same manner as cocaine and heroin within the three workflows analysed. The worsening of the molecular maps following BY40 staining may be due to a) strong absorption of the dye in the UV region, leaving little laser energy for the analytes present in the mark and b) depletion of the species of interest following immersion of the mark in stain and subsequent washing of the excess stain with deionised water. In addition, in all cases where a poor image or no image was obtained, post-imaging MS and MS/MS analysis revealed and confirmed the presence of the drug of abuse. These post-imaging analyses show the opportunity to provide intelligence on the presence of these substances, which can be very helpful, especially if CAF yielded a suitable ridge pattern image. Furthermore, these analyses show that MALDI is only a partially destructive process as the chemistry of the mark (and the ridge detail) can still be interrogated. Generally, though in our experiments the acetone treatment post-CAF development did not seem to yield an improvement in the detection of the ion signals, which is different than previously reported^11^, it was possible to obtain a fingermark ridge pattern image for cocaine (in addition to heroin and cholesterol). This shows that acetone post treatment is not required for the detection of these drugs under the experimental and instrumental conditions described in this paper.

In experiment set B, detection and mapping of the same four drugs and endogenous species by MALDI was investigated after the following enhancement scenarios: (i) VMD, (ii) CAF + VMD and (iii) CAF + BY40 staining + VMD. Interestingly, in the majority of cases, the application of VMD boosted the ion signal, as reflected by stronger MALDI images compared to the corresponding undeveloped marks (Fig. 5B), which corroborates previous observations^5^. The comparison between undeveloped and VMD-developed marks shows that amphetamine, cocaine, THC and cholesterol provide more intense molecular images after VMD development, whereas heroin appears to yield an equally strong image. In this scenario, satisfactory ridge detail was obtained for only cholesterol and heroin, whereas cocaine yielded a grade 2 image, which is more useful than that provided by the VMD optical image in this instance (over-developed, most likely due to the use of fresh fingermarks). Under enhancement scenario (ii), all species, including amphetamine and THC, provide a suitable signal accompanied by ridge detail in the undeveloped half of the mark. The enhancement of the other half of the fingermark by CAF followed by VMD also yields a robust signal, which is stronger for cocaine and heroin. This enhancement affected the mapping of amphetamine, THC and cholesterol, as their images showed scarce ridge detail. Finally, under scenario (iii), all species exhibited very strong signals again, providing excellent ridge detail in the undeveloped halves of the mark. The corresponding fingermark halves treated with CAF followed by BY40 staining and VMD yielded less intense signals and reduced ridge continuity. However, in this case, an increase in brightness, to the point of over-saturating the image of the undeveloped half of the mark, showed usable ridge detail generated by mapping of cocaine, heroin and endogenous cholesterol. As with experiment set A, in set B, the presence and identity of the species were confirmed by post-imaging MS and MS/MS analyses.

## Conclusions

For the first time, MALDI profiling (MS and MS/MS) and imaging (in MS mode) protocols for the detection, confirmation, and mapping of a large range of drugs and their metabolites in fingermarks, as well as for determining the limits of detection (sensitivity), have been investigated and optimised. This work was aimed at furthering knowledge regarding the compatibility of MALDI with currently used forensic enhancement techniques (FET), in particular with cyanoacrylate fuming (CAF) and Vacuum Metal Deposition (VMD). Prior chemical treatment of the marks was generally expected to have an effect on the ability of MALDI to yield the desired ion signal and corresponding 2D visualisation within the mark^5^,^8^ (as was also observed in this work). Nonetheless, we have shown here that prior application of FET does not prevent the detection of forensically interesting species, such as illicit drugs and their metabolites. Furthermore, molecular maps were retrieved after CAF and VMD enhancement. Treatment with acetone following CAF did not improve the ion signal; however, in contrast to work published by others, 2D maps depicting the presence of cocaine on the mark ridges were nevertheless obtained. In the present study, it was observed that although the quality of the ridge pattern was strongly molecule dependent, VMD treatment appeared to be much more compatible than CAF, confirming the general ability of gold to enhance the MALDI signal. Although more work is needed to extend these studies to additional drugs and metabolites in “natural” fingermarks (e.g., those obtained with no prior fingertip cleaning or grooming procedure) deposited on a range of CAF and VMD amenable surfaces, the data presented here confirm the strong potential for implementing MALDI technology within the fingermark enhancement and chemical mapping workflows routinely used in forensic casework.

## Additional Information

**How to cite this article**: Groeneveld, G. *et al.* Detection and mapping of illicit drugs and their metabolites in fingermarks by MALDI MS and compatibility with forensic techniques. *Sci. Rep.* **5**, 11716; doi: 10.1038/srep11716 (2015).

## Supplementary Material

Supplementary Information

## Figures and Tables

**Table 1:** List of the *m/z* values detected for drugs, their metabolites and their associated ion fragments via MS and MS/MS as well as the limits of detection observed in the two MALDI imaging experiments (spotted drug and high/low concentration spiking).

**Drug**	**MALDI–QTOF–MS**		**MALDI-QTOF-MS/MS**		**MALDI-QTOF-MSI**			
	Observed [M+H]^+^ & IS fragment	Difference (amu)*	*m/z* precursor →productions**	Ref	LOD ng/μL	LOD*** ng/cm^2^	#Handling ng/cm^2^	#Abuse ng/cm^2^
Amphetamine C_9_H_13_N C_9_H_13_N	136.1371 [M+H]^+^ 91.0751 [ISF]	–0.0245	136 → 119, 91	24	10	2,96	189.5	18,95
4–MeAmph C_10_H_15_N	150.1315 [M+H]^+^	–0.0032	150 → 133, 105, 91	24	−	−	−	−
MethAmph C_10_H_15_N	150.1315 [M+H]^+^	–0.0032	150 → 119, 91	[24, 25]	−	−	−	−
MDMA C_11_H_15_NO_2_	194.1458 [M+H]^+^ 163.1017 [ISF]	–0.0277	194 → 163, 135,105,58	[24, 25]	1	0,88	189.5	18,95
MDA C_10_H_13_NO_2_	180.1367 [M+H]^+^ 163.1015 [ISF]	–0.0343	180 → 163, 135,105,72	[24, 25]	1	0,88	189.5	
MDEA C_12_H_17_NO_2_	208.1664 [M+H]^+^ 182.1474 [ISF]	–0.0327	208 → 163, 135, 105, 72	[24, 26]	−	−	−	−
Cocaine C_17_H_21_NO_4_	304.1907 [M+H]^+^ 182.1474[ISF]	–0.0359	304 → 272, 182, 150, 82	[24, 26]	0,01	0,0088	189.5	0.19
BZE C_16_H_19_NO_4_	290.1708 [M+H]^+^ 168.1270 [ISF]	–0.0316	290 → 272, 168, 150,82	[24, 26]	0,1	0,088	189.5	1,895
EME C_10_H_17_NO_3_	200.1548 [M+H]^+^ 182.1495 [ISF]	–0.0262	200 → 182, 168, 150,82	26	0,1	0,088	189.5	1,895
Methadone C_21_H_27_NO	310.2474 [M+H]^+^ 265.1706 [ISF]	–0.0303	310 → 265, 247, 223,159,105	24	−	−	−	−
Heroin C_21_H_23_NO_5_	370.1841 [M+H]^+^ 310.1846 [ISF]	–0.0187	370 → 328, 310, 286,211,193	[24, 27]	0,1	0,088	189.5	1,895
6–MAM C_19_H_21_NO_4_	328.1948 [M+H]^+^ 268.1733 [ISF]	–0.0400	328 → 310, 286, 211,193,165	[24, 27]	0,1	0,088	189.5	1,895
Morphine C_17_H_19_NO_3_	286.1737 [M+H]^+^	–0.0294	286 → 268, 211, 193,165	[24, 27]	0,1	0,088	189.5	1,895
THC C_21_H_30_O_2_	315.2712 [M+H]^+^	–0.0389	315 → 315, 300, 259,193,135,123	24	10	2,96	189.5	1,895
THCA C_21_H_28_O_4_	345.2463 [M+H]^+^	–0.0397	345 → 327, 299, 257,229,193	24	10	2,96	189.5	1,895
2 CB C_10_H_14_^79^BrNO_2_	260.0612	0.0326		28	−	−	−	−
C_10_H_14_^81^BrNO_2_	262.0647	0.0381	262 → 245, 230, 164					
mCPP C_10_H_13_ClN_2_	197.0926 [M+H]^+^	–0.0075	197 → 181, 154, 119,91	28	−	−	−	−

The table also reports MS/MS conditions and the literature references supporting structural confirmation and molecular identification.

^*^Calculated difference between measured *m/z* and calculated monoisotopic [M+H]^+^.

^**^MS/MS product ions obtained for structure elucidation, supported by previous studies.

^***^ng/cm^2^ as determined by dividing the amount of drug (volume/concentration) over the area of the applied spot (cm^2^) as shown in (see Fig. 3).

^#^ng/cm^2^ as determined by dividing the amount of drug (pure amount) over the area of a fingermark (~2.64 cm^2^) (see Fig. 4).
