# Supplementary material for: Detection and mapping of illicit drugs and their metabolites in fingermarks by MALDI MS and compatibility with forensic techniques
Source: Sci Rep. 2015 Jun 29;5:11716. doi: 10.1038/srep11716 (PMC4484357; doi:10.1038/srep11716)
Supplement: Supplementary Information [file srep11716-s1.pdf]

Detection and mapping of illicit drugs and metabolites in fingermarks by MALDI MS and compatibility with forensic techniques

G. Groeneveld[1,2±], M. de Puit[1±], S. Bleay[3], R. Bradshaw[2] and S. Francese[2*]

[1] Department of Fingerprint Research, Netherlands Forensic Institute, The Hague, Netherlands

[2] Biomedical Research Centre, Sheffield Hallam University, Sheffield, UK

[3] Centre for Applied Science and technology, Home Office, Sandridge, UK

±These authors have equally contributed to the work being presented here

*Corresponding author, Dr Simona Francese, s.francese@shu.ac.uk

Supplemental material

Supplemental Figures

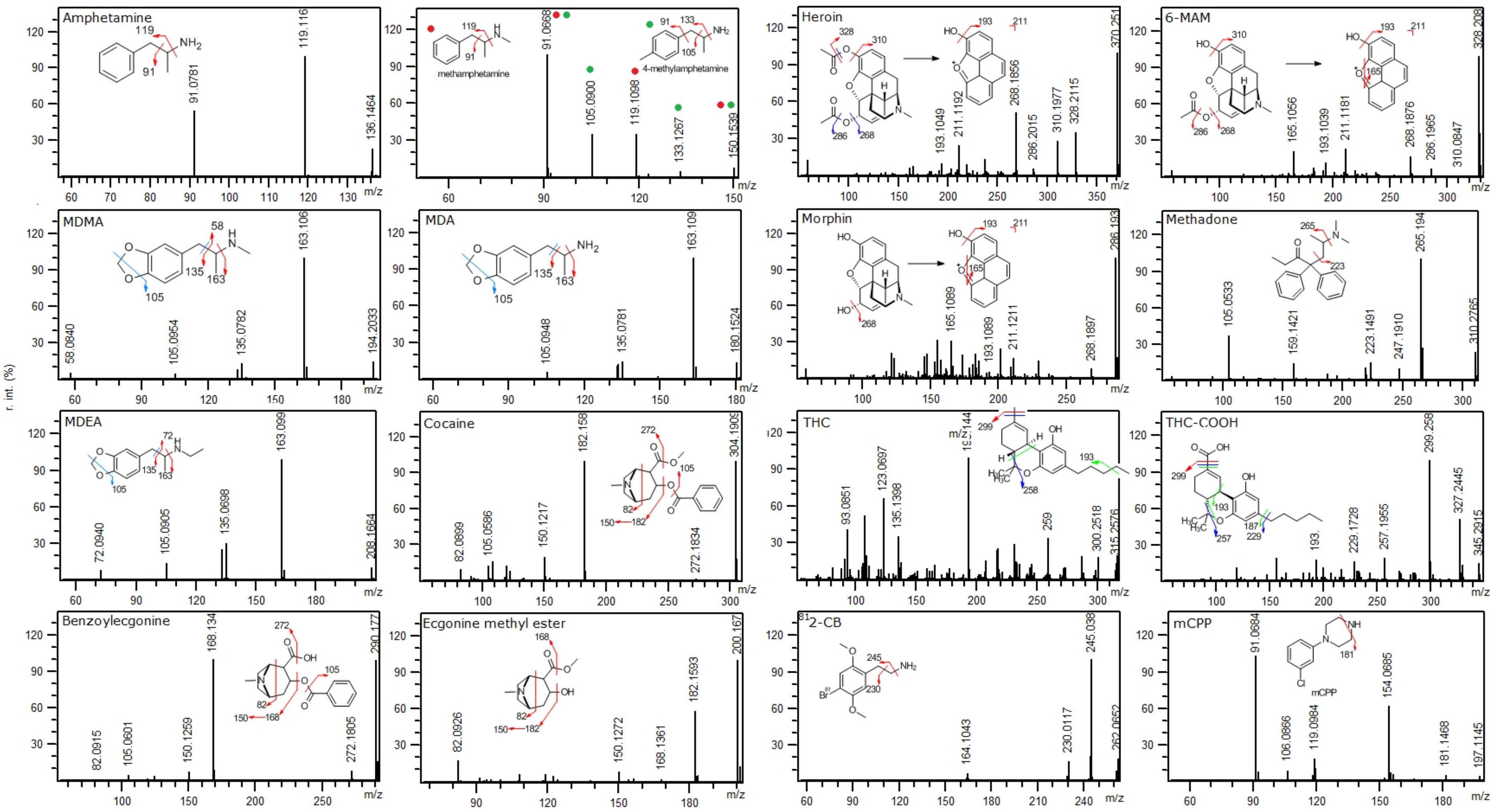

Fig. S1. MALDI MS/MS spectra of drug/metabolite precursor ions, showing distinct fragmentation pathways. The proposed fragmentation pattern and corresponding structures are shown. Red arrows indicate the one-step fragmentation of the parent ion, while blue and green arrows show two and three-step fragmentation respectively (progressive fragmentation of a fragmented ion).

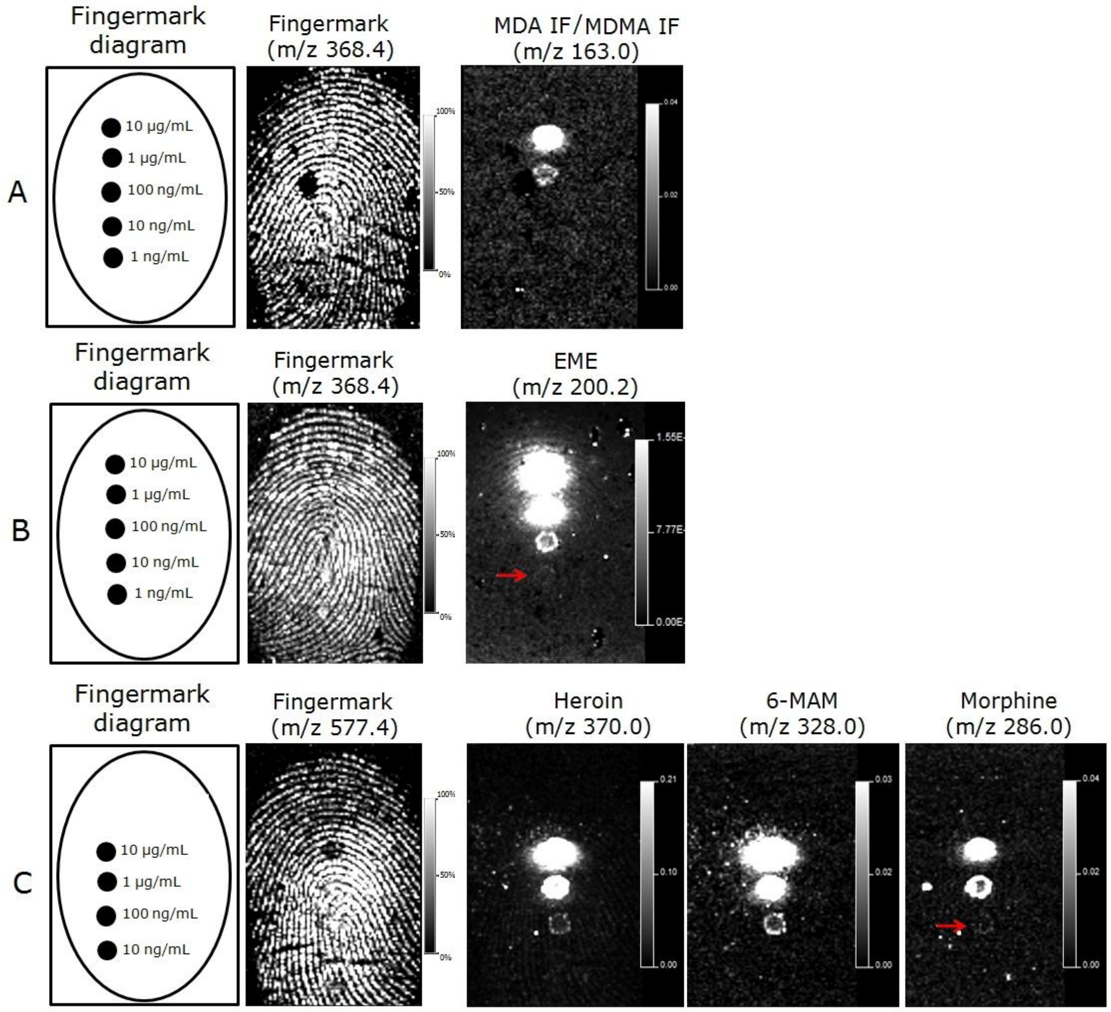

Fig. S2. Reprocessing of the data shown in Figure 3 and illustrating MALDI MSI data of drug classes serial dilutions ranging from 10 µg/mL – 1 ng/mL, spotted on top of a fingermark and subsequently spray-coated. Here images have been brightness-saturated to investigate if additional concentration spots were visible. Images were preliminarily normalised to the matrix peak at m/z 190. Out of all the species investigated, only MDA/MDMA, EME, Heroin, 6-MAM and morphin showed additional concentration spots with respect to Fig 3. In particular, with this reprocessing, MDA/MDMA becomes visible also in the spot at a concentration of 1µg/mL, whereas the remaining species could be mapped in the spots down to 100 ng/mL in concentration.
